# Breaking down the cellular responses to type I interferon neurotoxicity in the brain

**DOI:** 10.3389/fimmu.2023.1110593

**Published:** 2023-02-03

**Authors:** Barney Viengkhou, Markus J. Hofer

**Affiliations:** School of Life and Environmental Sciences and the Charles Perkins Centre, The University of Sydney, Sydney, NSW, Australia

**Keywords:** type I interferons, cerebral interferonopathies, neurotoxin, neurodegenerative diseases, aging, multiple sclerosis, Aicardi-Goutières syndrome, traumatic brain injury

## Abstract

Since their original discovery, type I interferons (IFN-Is) have been closely associated with antiviral immune responses. However, their biological functions go far beyond this role, with balanced IFN-I activity being critical to maintain cellular and tissue homeostasis. Recent findings have uncovered a darker side of IFN-Is whereby chronically elevated levels induce devastating neuroinflammatory and neurodegenerative pathologies. The underlying causes of these ‘interferonopathies’ are diverse and include monogenetic syndromes, autoimmune disorders, as well as chronic infections. The prominent involvement of the CNS in these disorders indicates a particular susceptibility of brain cells to IFN-I toxicity. Here we will discuss the current knowledge of how IFN-Is mediate neurotoxicity in the brain by analyzing the cell-type specific responses to IFN-Is in the CNS, and secondly, by exploring the spectrum of neurological disorders arising from increased IFN-Is. Understanding the nature of IFN-I neurotoxicity is a crucial and fundamental step towards development of new therapeutic strategies for interferonopathies.

## Introduction

Central nervous system (CNS) inflammation is involved in a wide range of neurological disorders and diseases, from pathogen-driven encephalitis and autoimmune disorders to trauma, aging, and neurodegeneration (1–4). The complex nature of inflammation is typically portrayed as either beneficial, such as pathogen elimination, or detrimental, like induction of cell death. Yet in many cases, these processes occur simultaneously and are driven by multiple mediators. The type I interferons (IFN-Is) are master regulators of inflammation. They include the IFN-α subtypes and IFN-β and were originally identified due to their ability to interfere with viral replication (5). However, a vast amount of research over the past 60 years has revealed that IFN-Is have a wide range of roles in addition to regulating inflammation and immunity.

There are three main mechanisms by which IFN-I production and signaling can be increased. Firstly, activation of innate immune sensors by pathogens or cellular danger signals triggers increased expression of IFN-I genes. For example, cytosolic dsDNA from viruses, damaged mitochondria, or improperly processed self-nucleic acids are recognized by cyclic GMP–AMP synthase (cGAS), which in turn activates the stimulator of interferon genes (STING) (6). Activated STING then triggers a signaling cascade resulting in the upregulation of IFN-I expression (6). In addition to STING, there are multiple other immune sensors that upregulate IFN-I expression in similar ways (7, 8). Secondly, genetic changes can result in increased IFN-I signaling such as in trisomy 21 due to an extra copy of IFN-I receptor 1 (IFNAR1) (9), or reduced negative regulation of the IFN-I pathway such as in patients with mutations in *USP18* or *ISG15* (10, 11). Thirdly, IFN-Is are used as treatment for a range of diseases including chronic viral infections (12), multiple sclerosis (MS), and several cancers and tumors (13–16).

**Figure 1:**
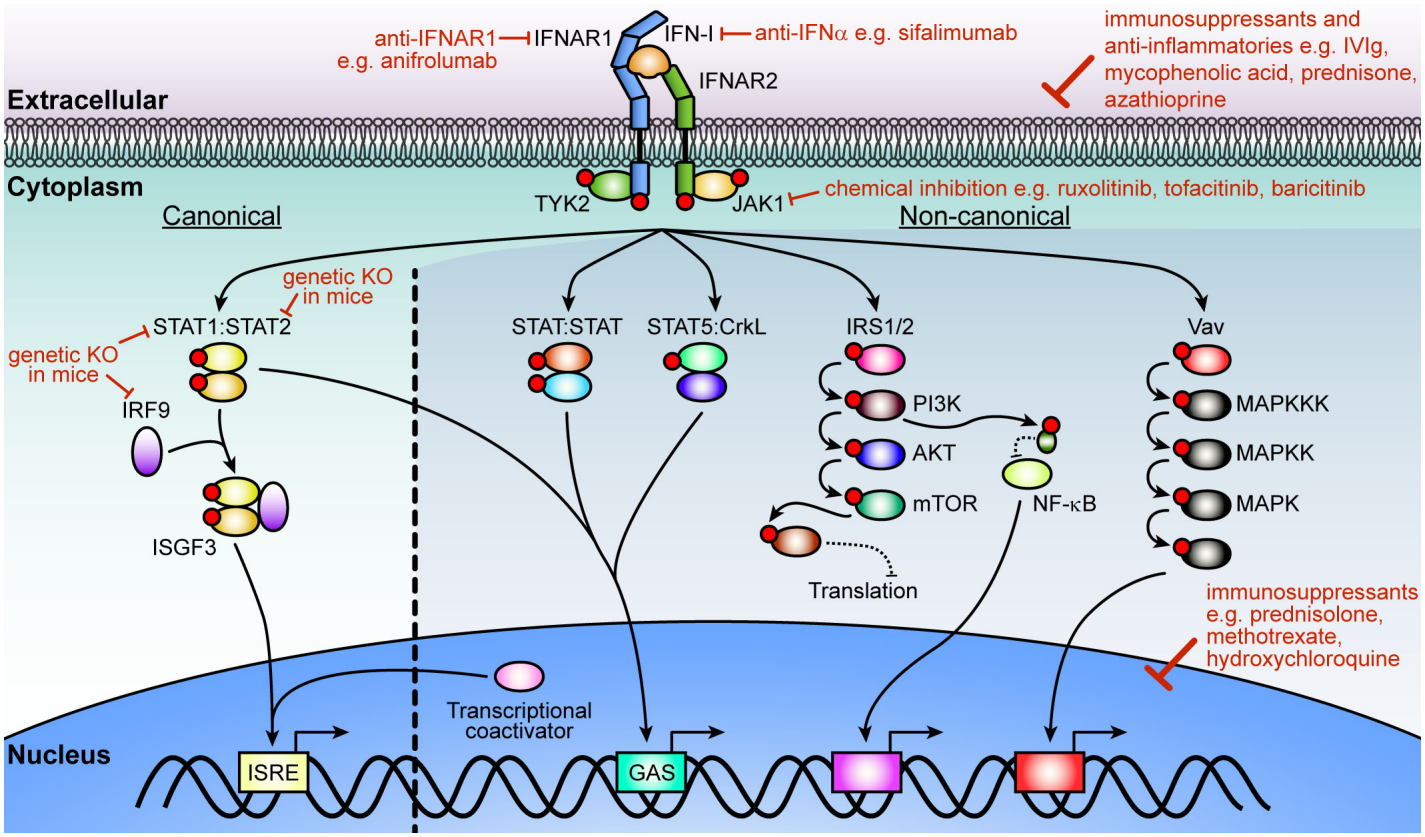
IFN-I signaling pathway and strategies of inhibition. After ligation of IFN-Is with its cognate receptor chains, IFNAR1 and IFNAR2, JAK1 and TYK2 transphosphorylate each other before phosphorylating the receptors. In the canonical pathway, STAT1 and STAT2 dock at the receptor to become phosphorylated by the JAKs. Phosphorylated STAT1 and STAT2 then form a trimolecular complex (ISGF3) with IRF9 and translocate into the nucleus to bind ISREs to regulate the expression of hundreds of interferon-regulated genes (IRGs). Non-canonical signaling involves homodimers or heterodimers of STATs, STAT5 binding to CrkL, or recruitment of transcriptional coactivators to regulate ISRE or GAS elements. Additional kinases are activated (PI3K, NF-κB and MAPK pathways) which modulate the cellular response to IFN-Is that includes translation of a subset of genes, regulation of transcription or a range of cellular functions. Multiple strategies have been employed to target IFN-I signaling including inhibition or elimination of proteins in the pathway or its overall effects with immunosuppressants and anti-inflammatories that act on the cell or affect the expression of genes associated with inflammation. Red circles indicate phosphorylation of a protein. IFN-I, type I interferon; IFNAR, IFN-α/β receptor; JAK1, Janus kinase 1; TYK, tyrosine kinase 2; STAT, signal transducer and activator of transcription; IRF9, interferon regulatory factor 9; ISFG3, interferon-stimulated gene factor; ISRE, interferon-stimulated response-elements; GAS, γ-activated sequence; CrkL, Crk like proto-oncogene, adaptor protein; IRS, insulin receptor substrate; PI3K, phosphoinositide 3-kinase; mTOR, mammalian target of rapamycin; NF-κB, nuclear factor-κB; MAPK, mitogen-activated protein kinase.

All IFN-Is mediate their cellular effects through binding to a single heterodimeric cell surface receptor consisting of the IFNAR1 and IFNAR2 chains. Activation of the receptor complex triggers two distinct signaling phases (**Figure 1**). The first phase induces rapid and widespread changes to protein phosphorylation and affects multiple signaling pathways including mitogen-activated protein kinase, cyclin-dependent kinase, and AKT (17). While still not fully understood, it appears that this widespread change in protein phosphorylation prepares the cell for the second phase, which modulates the expression of several hundreds of IFN-regulated genes (IRGs). To make matters more complex, this transcriptional phase mediates its effects through several signaling pathways. Of these, the best understood is the activation of the interferon-stimulated gene factor 3 (ISGF3) complex, which consists of the transcription factors signal transducer and activator of transcription (STAT1) 1, STAT2, and interferon regulatory factor 9 (IRF9). The ISGF3 pathway is often also called the canonical IFN-I signaling pathway and is critical to activate the antiviral response. By contrast, all other pathways are termed ‘non-canonical’ and are thought to modulate the antiviral response in a cell- and stimulus-dependent context (18–22). Moreover, the signaling components in the IFN-I pathway and can be activated by other cytokines, which complicates defining the precise contribution of IFN-Is in inflammation and immunity *in vivo*. In particular, while IFN-IIIs bind to their unique cell surface receptor, they also mediate their effects through the ISGF3 complex. Recent findings suggest that IFN-IIIs, which consist of the IFN-λs, contribute to neuroinflammation, however, many aspects remain unclear. It appears that IFN-Is are more potent than IFN-IIIs (23, 24) and that the expression of the IFN-III receptor is restricted (25) with very low transcript levels in the brain (23, 24). Thus, while we will not discuss the role of IFN-IIIs in detail, it is important to keep in mind that synergism and antagonism of signaling pathways between IFN-Is and other cytokines influences the outcomes of IFN-I-induced cellular and tissue responses.

Although IFN-Is are critical for the physiological regulation of inflammation, they are associated with a range of adverse effects. These adverse effects manifest often as neurological deficits and are commonly observed when IFN-Is are used as a drug or in patients with chronically elevated IFN-I production in the brain (26). Importantly, the cellular and molecular basis for this IFN-I neurotoxicity remains unclear and its study is complicated by the presence of multiple cell types in the CNS (e.g., neurons, glia, and vascular cells), each of which shows unique cell type-specific responses (17, 27–30). Accordingly, in this review, we dissect the complexity of IFN-I neurotoxicity at two levels: firstly, by analyzing the cell-type specific responses to IFN-I in the CNS, and secondly, exploring the spectrum of diseases and symptoms of neurological disorders with increased IFN-Is.

## Cellular responses to IFN-Is in the brain

The existence of a homeostatic level of IFN-I signaling in the brain is demonstrated by the presence of IRG products in the healthy brain (31, 32) and reduced expression of IRGs in unstimulated IFNAR1-deficient mice (18) and cells lacking IFN-I signaling proteins (33). The role of homeostatic IFN-I signaling in the brain is diverse and ranges from priming cells for detection and response to pathogens to roles in learning and memory. For example, several studies have shown that neutralization of IFNAR1 results in synapse reduction and impaired synaptic plasticity (34) and ablation of IFN-β leads to defective neuronal autophagy (35). In addition to homeostatic production, IFN-I expression can be markedly increased in most if not all brain-resident cells in response to a range of stimuli. Recent progress in omic analyzes, particularly at the single-cell level, has demonstrated that within the diseased brain, a spectrum of cellular response states occurs simultaneously rather than a uniform response (36–42). Moreover, while all cell types in the CNS can respond to IFN-Is, each cell type mounts its specific response to IFN-Is. Consequently, the sum of the individual responses determines the local tissue response. In the following sections, we will summarize these cell-type specific responses.

### Neurons

Neurons require IFN-I signaling for normal development. Homeostatic IFN-β signaling in neurons is involved in the formation of dendritic spines, neurite branching, and neuronal autophagy, while loss of IFNAR1 signaling in neurons leads to formation of protein aggregates or Lewy bodies (35). However, IFN-β injected into the brain also causes a reduction of synapses (43), demonstrating the importance of balanced IFN-I signaling for neuronal function. In response to viral infections, neurons show limited production of IFN-Is (44, 45).

Importantly, while they mount a robust response to IFN-Is, neurons only regulate the expression of a limited set of IRGs (46, 47). This comparatively (to other CNS cell types - see below) narrow response provides antiviral protection and may serve to limit adverse or detrimental effects of IFN-I signaling in these delicate cells. The need to protect neurons from damage is also supported by the elevated basal expression of some IRGs like *ISG15* in neurons compared with other cells contributing to an intrinsic antiviral resistance (48). IFN-I mediated neurotoxicity manifests in neurons after IFN-α treatment with fewer dendrites (49, 50), decreased neuronal neurogenesis (51), reduced neurotrophic signaling (52), and increased apoptosis of precursor cells (53). In addition, IFN-α alters glutamate-induced excitatory potentials in hippocampal neurons and inhibitory post synaptic potentials in pyramidal neurons (47, 54–56). This in turn may increase epileptiform discharges associated with seizures and inhibit long term potentiation, a process important in memory formation (47, 54–56). Moreover, antagonizing the glutamate receptor, N-methyl D-aspartate receptor (NMDAR), reduces the neurotoxicity of IFN-α, indicating a toxic role of IFNAR and NMDAR coactivation (50). IFN-β also modulates ion channels to increase the number of action potentials elicited after activation of protein kinase C (56) and is in line with IFN-β altering glutamatergic neurotransmission (57). In addition, increased cerebral IFN-α levels in transgenic mice with CNS-targeted overproduction of IFN-α (termed GFAP-IFN mice) results in a progressive loss of neurons (58), impaired learning (59), and changes in phosphoproteins that are associated with various neuronal functions (17). Thus, increased IFN-I signaling has detrimental effects on neuronal health and survival.

### Astrocytes

Astrocytes are the most abundant glia cell and tile the CNS. Similar to neurons, basal IFN-I signaling in astrocytes is required for a healthy brain. Astrocyte-specific deletion of IFNAR1 results in impaired learning, reduced synapse plasticity, and fewer synapses (34). Following infection with neurotropic viruses, astrocytes are the main producers of IFN-β in mice (44, 60). Their response to IFN-Is is required to limit pathogen replication (61) and to promote blood–brain barrier (BBB) integrity following virus infection (23). Astrocytes alter morphology in response to IFN-Is as observed in brains of patients with increased cerebral IFN-I production (62–64) and GFAP-IFN mice (17, 59). Treatment of astrocytes with IFN-α or IFN-β reduces astrocytic process complexity and domain range and also upregulates genes involved in antiviral responses, metabolism, apoptosis, and major histocompatibility complex (MHC) (17, 27, 39, 59, 62–64). Of note, increased levels of MHC on astrocytes negatively impact neuronal function, activate microglia, and are correlated with social and cognitive deficits in mice (65). Astrocytes can facilitate leukocyte infiltration by increasing chemokine expression after IFN-α treatment (66). In line with this, a subset of astrocytes located around outer cortical blood vessels, and thought to regulate leukocyte access, has been identified as being highly responsive to IFN-Is (39). This highly IFN-I-responsive subset has also been identified in mouse models of Alzheimer’s disease (AD), MS, and acute cortical trauma (39). Hypertrophic astrocytes and increased parenchymal leukocytes are also observed in brains of GFAP-IFN mice, supporting a role for astrocytes in mediating leukocyte infiltration (58, 59). While these findings suggest an inflammation-promoting role of IFN-Is on astrocytes, IFN-I signaling in astrocytes can also limit neuroinflammation through the production of the aryl hydrocarbon receptor and suppressor of cytokine signaling 2, dampening activation of proinflammatory signaling pathways (67). Specifically, mice with astrocyte-restricted *Ifnar1*-knockdown show exaggerated neuroinflammation in experimental autoimmune encephalomyelitis (EAE), a mouse model of MS (67). In addition, IFN-α but not IFN-β treatment of human astrocytes reduces proliferation and glucose uptake (68) which impacts the metabolic heath of the CNS. Thus, while the contribution of astrocytes to IFN-I neurotoxicity is not clear, these findings suggest a complex role for astrocytes in modulating IFN-I responses, one that is of increasing interest.

### Microglia

Unlike neurons or astrocytes, microglia do not originate from the neuroectoderm. They are derived from the yolk sac and colonize the brain early during embryonic development (69). Microglia are highly plastic and sensitive to the local environment and are considered the key immunoresponsive cell type in the CNS. Microglia produce IFN-α and IFN-β in a wide range of neurological diseases ranging from viral infection to autoimmune disorders (44, 70, 71). Microglia show a more rapid and diverse response to IFN-α compared with astrocytes and neurons (17, 27, 46). Similar to astrocytes, microglia morphology has been used as an indicator of their functional state (72). However, rather than changing into an amoeboid morphology, which is typically observed of microglia in inflammatory situations, in response to IFN-Is, microglia become hyper-ramified with increased process complexity (73). This is also observed in AD and aging (74), indicating microglia are responding to IFN-Is in these conditions. In response to IFN-α, microglia upregulate expression of IRGs, cytokines and chemokines and increase antigen presentation (27), enabling them to act as antigen-presenting cells, propagate inflammation, and promote leukocyte infiltration. This transcriptomic response has been similarly identified in microglia in the aged brain, AD or demyelination in humans or mouse models (38, 40–42). Although most microglia upregulate IRGs, there is a small subset of microglia that are IFN-I-hyperresponsive as identified by single-cell sequencing of a large number of microglia (36, 38, 40, 41). It has been suggested that this hyperresponsive subset contributes to age-dependent cognitive decline and increased synaptic stripping (75–77). In support, minocycline inhibition of microglia activation reduced features of depression and impaired learning of fear extinction in mice injected with IFN-α (78) and use of anti-IFNAR1 treatment in a mouse model of AD demonstrated that IFN-Is promote microglial engulfment of synapses (79). Additionally, minocycline has been used in various neurodegenerative diseases with varied outcomes in animal and human studies (80). However, a recent study using GFAP-IFN mice has demonstrated that depletion of microglia exaggerated disease (81), suggesting that the role of these cells in IFN-I-driven disease may be both beneficial and detrimental.

### Oligodendrocytes

Oligodendrocytes have limited responses to IFN-α and IFN-β. In viral infections, oligodendrocytes have low production of IFN-Is and show less expression of IRGs, compared with microglia (82). Additionally, IFN-α or IFN-β have no effect on oligodendrocyte proliferation or survival (31, 51, 83). This suggests on the one hand a partial refractory state of oligodendrocytes to IFN-Is, and on the other hand, that the loss of myelin in neurodegenerative diseases may be an indirect response due to actions from surrounding cells or other mediators rather directly through IFN-I signaling. In support of this, a study using single-cell transcriptomics in a mouse model for MS identified a subset of oligodendrocytes that actively recruit T cells, driving the loss of myelin (37). However, data on oligodendrocyte responses to IFN-Is remains limited and further studies are needed to provide a deeper understanding how IFN-Is affect these cells.

### Blood–brain barrier and endothelial cells

The BBB is critical for maintaining CNS homeostasis and brain function (84) and plays crucial roles in neuroinflammation by regulating the migration of leukocytes and diffusion of plasma proteins into the brain parenchyma (85). This separation between blood and brain tissue differs from most other vascular barriers, resulting in vascular cells of the BBB adopting a comparatively distinct phenotype (86). The vascular cells forming the BBB include endothelial cells, pericytes, and mural cells. In particular, cerebral endothelial cells may contribute more to IFN-I signaling in the murine CNS than other cell types as single-cell transcriptomics indicate expression of *Ifnar1* and *Ifnar2* is higher in these cells than in microglia, astrocytes, and neurons (87, 88). Similarly, in humans, *IFNAR2* expression is higher in endothelial cells than glia and neurons (89). This responsiveness of the vasculature is also evident from reports of systemic vasculitis and loss of BBB integrity in patients receiving IFN-Is (55, 90, 91). This vasculopathy is amplified in patients with cerebral interferonopathies and in GFAP-IFN mice, where aneurysms and perivascular calcification are hallmarks of the disease (58, 62, 63, 91). However, the mechanisms leading to these pathologies are unclear, and studies suggest opposing actions of IFN-Is. IFN-α blocks angiogenesis and is toxic to endothelial progenitor cells, contributing to irregular vasculogenesis, abnormal repair and increased atherosclerosis (92). IFN-I therapy can also cause thrombotic microangiopathy and aneurysms (91). The response of endothelial cells in the BBB to IFN-β leads to the secretion of C-X-C motif chemokine 10 resulting in compromised neuronal function and sickness behavior (30). *In vitro* studies support the BBB-damaging effects of IFN-Is, showing that IFN-α and IFN-β enhance endothelial apoptosis and reduce angiogenesis (93–96). Yet, other studies found that IFN-α induces endothelial proliferation (97, 98) and that IFN-β signaling in endothelial cells has anti-inflammatory roles by inhibiting intracellular signaling of proinflammatory pathways and promoting BBB integrity in the host response to viruses and in MS (23, 99, 100). While the basis for these reported differences in endothelial responses to IFN-Is remains unclear, it points to the importance of the subtype of IFN-Is involved and also the context in which IFN-I signaling occurred. Nevertheless, the impact of IFN-Is on the cerebral vasculature has an active role in disease progression of patients with cerebral interferonopathies and in other neurodegenerative diseases. Accordingly, should further studies demonstrate a direct pathogenic role for the brain’s vasculature, this would open new therapeutic avenues as in contrast to the brain’s parenchyma, the vessels are easily targeted by peripheral drugs.

## Neurological disorders with increased IFN-I

**Figure 2:**
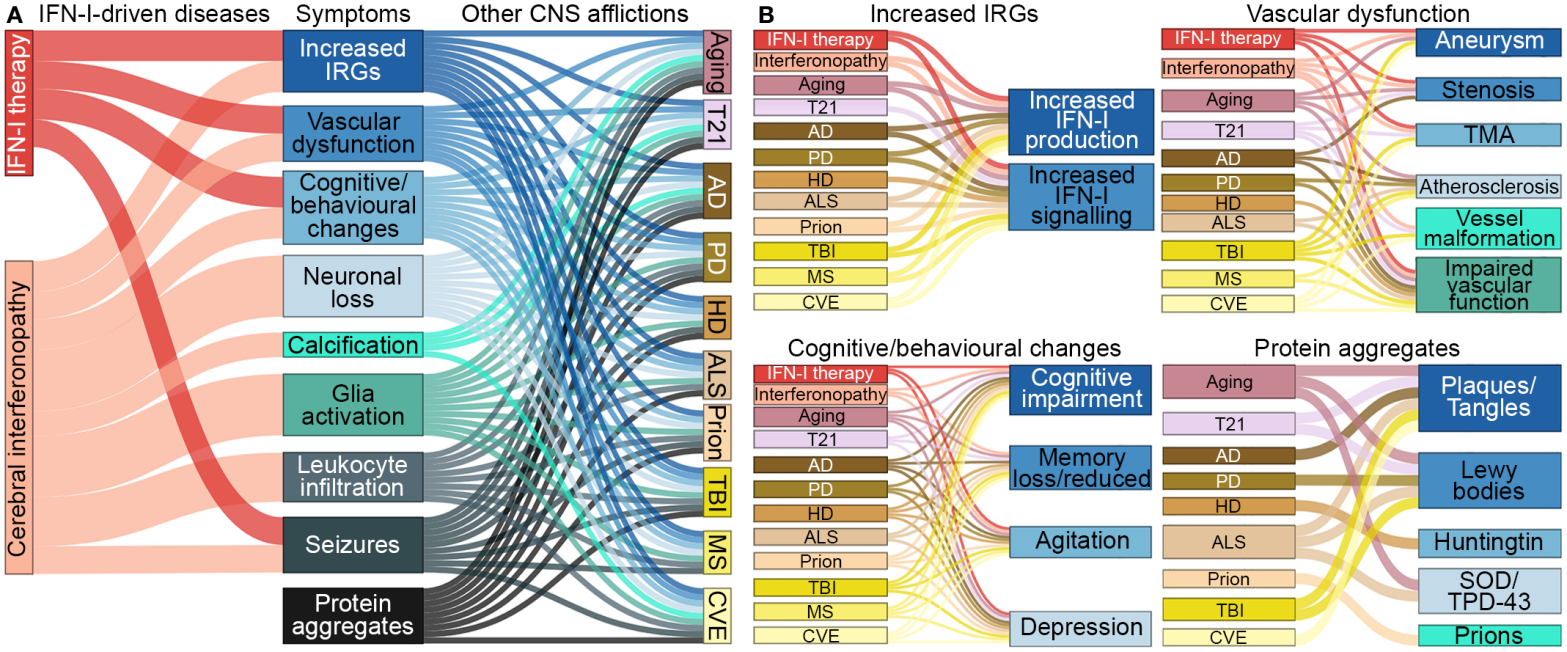
Symptomatic links between IFN-I-driven diseases and CNS afflictions. **(A)** Symptoms that arise in diseases driven by IFN-Is overlap with symptoms that occur in aging, trisomy 21 and several neurodegenerative diseases, trauma, autoimmune diseases and chronic viral infections, CNS afflictions found to have increased IFN-I signaling. **(B)** Further breakdown of symptoms linked to each of the CNS afflictions. Note, protein aggregates can lead to increased IFN-Is and expression of IRGs. CNS-centric symptoms were compared and linked if there was prevalence in several human cases. Size of nodes and links are arbitrary. T21, Trisomy 21; AD, Alzheimer’s disease; PD, Parkinson’s disease; HD, Huntington’s disease; ALS, amyotrophic lateral sclerosis; TBI, traumatic brain injury; MS, multiple sclerosis; CVE, chronic viral encephalopathy; TMA, thrombotic microangiopathy; SOD, superoxide dismutase; TPD-43, transactive response DNA binding protein 43 kDa.

There is growing evidence that inflammatory processes and, in particular, IFN-Is, are involved in a wide range of neurological diseases (**Table 1**) (1–3). The symptomatic overlap between these diseases, as well as the reported adverse effects of IFN-I therapy, suggests a causal contribution of increased IFN-I signaling to their pathogenesis (**Figure 2**). However, the specific contribution of IFN-Is to the pathogenesis of these diseases is often not well understood.

**Table 1:** IFN-I signaling and its inhibition in neurological disorders.

Disorder	Cause of increased IFN-I signaling	Blocked IFN-I induction or signaling	Consequence
Aicardi-Goutières syndrome (AGS)	Genetic mutation in genes associated with nucleic acid regulation	JAK inhibitor	(H) Improvement of symptoms including some neurologic features (101–107)
USP18 deficiency	Mutation in *USP18* that reduces negative regulation of IFN-I signaling	JAK inhibitor	(H) Remission of symptoms (10)
		USP18^-/-^ x IFNAR1^-/-^ mice	(M) Normal phenotype (31)
Systemic lupus erythematosus (SLE)	Unknown	Anti-IFNAR1	(H) Improvements of some symptoms (108, 109) (M) No change phenotypic change (110) (M) Rescue of some autoimmunity features, no change, or worsen survival dependent on model (111)
		Anti-IFN-α	(H) Improvements of symptoms (112)
		JAK inhibitor	(H) Improvements of some symptoms (113)
		x IFNAR^-/-^ mice	(M) Attenuated disease phenotype (114–117)
Chronic viral encephalopathy	Chronic response to viruses	IFNAR1 deficiency	(H) Lethal infection (118)
Aging	Unknown	Anti-IFNAR1	(M) Improved cognitive function, reduced gliosis, and reduced age-related neuroinflammation (119)
		JAK inhibitor	(M) Improved physical functions and coordination (120)
Trisomy 21	Increased expression of IFNAR	JAK inhibitor	(H) Improvement in peripheral symptoms, central symptoms not reported (121–123) (M) Improved survival and reduced loss of weight when immunologically challenged (124)
Alzheimer’s disease (AD)	Microglia response to nucleic acid containing plaques (43)	Anti-IFNAR	(M) Restored microglia activity (43) (M) Rescued cognitive function (79)
		APP_SWE_/PS1_ΔE9_ x IFNAR1^-/-^	(M) Reduced cognitive decline and anti-inflammatory glia response (125)
Parkinson’s disease (PD)	α-synuclein aids in neuron-specific IFN-I responses (126)	MPTP-treated IFNAR1^-/-^ mice MPTP-treatment and anti-IFNAR1	(M) Reduced neuroinflammation and reduced loss of dopaminergic neurons (127)
Huntington’s disease (HD)	Activation of cGAS/STING which indues IFN-Is (128) Mutant huntingtin leads to mitochondrial dysfunction which induces IFN-Is (129)	cGAS deletion	(M) Reduced expression of proinflammatory genes and reduced autophagy (128)
Amyotrophic lateral sclerosis (ALS)	Accumulated TDP-43 activates cGAS/STING to induce IFN-Is (130)	SOD1 x IFNAR1^-/-^	(M) Prolonged survival (131)
		x STING^-/-^ mice STING inhibitor	(M) Reduced IFN-I gene expression, prevented loss of neurons, and improved motor function (130)
Prion	STING mediated IFN-I induction (132)	IFNAR1^-/-^ mice	(M) Reduced neuroinflammation and prolonged survival from slowed disease progression (132)
Traumatic brain injury (TBI)	STING-mediated IFN-I induction (4)	STING^-/-^ mice	(M) Reduced neuroinflammation, reduced lesion size, and completion of autophagy process (4)
		IFN-β^-/-^ mice	(M) Reduced proinflammatory response, improved motor and cognitive functions, and reduced neurodegeneration (133)
		Anti-IFNAR1	(M) Improved motor and cognitive functions and no change in lesion volume (133) (M) Reduced infarct volume, reduced inflammatory response, and improved behavioral outcomes (134)
		IFNAR1^-/-^ mice	(M) Reduced infarct volume and reduced inflammatory response (134)
Multiple sclerosis (MS)	Increased around lesions	EAE in IFNAR1^-/-^	(M) More severe disease, increased neuroinflammation, and increased demyelination (28)
		EAE in IFNAR1^-/-^ EAE in IFN-β^-/-^	(M) Increased myelin debris accumulation (71)
GFAP-IFN mice	Transgenic overproduction of IFN-α in the brain	x IFNAR1^-/-^ mice	(M) WT-like phenotype (91)
		x STAT1^-/-^ mice	(M) Exacerbated disease (91, 135)
		x STAT2^-/-^ mice	(M) Different disease pathology (135, 136)
		x IRF9^-/-^ mice	(M) Exacerbated disease (136, 137)

cGAS, cyclic GMP-AMP synthase; EAE, experimental autoimmune encephalomyelitis, a model for MS; IFN-I, type I interferon; IFNAR, type I interferon receptor; IRF, interferon regulatory factor; JAK, Janus kinase; MPTP, 1-methyl-4-phenyl-1,2,3,6-tetrahydropyridine, used to model PD; STAT, Signal transducer and activator of transcription; STING, Stimulator of interferon genes; USP18, Ubiquitin specific peptidase 18; WT, wild-type; (H) indicates findings in humans and (M) indicates findings in mice.

### Type I interferons directly induce neurotoxicity

The direct neurotoxic effects of IFN-Is are well documented due to their clinical use (53, 138–143). Common (>20%) adverse neurological reactions in patients include flu-like symptoms, fatigue, and depression. Less commonly (<5%) observed adverse events include personality changes, cognitive dysfunction, memory loss, mood disorders, psychomotor slowing, and rare (<1%) but severe reactions including psychosis, mania, and seizures. Nature and severity of adverse reactions is dose dependent and generally worsens over time. Fortunately, cessation of treatment leads to an eventual recovery in most cases (140), indicating that these reactions are mediated by IFN-Is rather than the underlying condition for which IFN-Is have been used as treatment. Importantly, the requirement of basal IFN-I signaling for normal brain development suggests a threshold above which IFN-Is become neurotoxic. This is further supported by findings in glioblastomas. In a subset of glioblastoma, stem cells that display elevated cell-intrinsic IFN-I signaling, which contributes to tumor growth, IFN-β treatment can induce cell death, but not in tumor stem cells that have lower cell-intrinsic IFN-I signaling (144, 145). Several mechanisms by which IFN-Is mediate neurotoxicity have been proposed. For example, IFN-α-induced neuropsychiatric symptoms have been associated with changes in glucose metabolism and neuronal circuitry activity in the basal ganglia and prefrontal cortex (146–148), decreased tryptophan availability with altered serotonergic signaling (149–152) and increased presence of proinflammatory cytokines (141, 149, 152–154). IFN-α treatment can also cause retinopathy (30–86% occurrence) (90, 155) and focal BBB leakage which potentially induces seizures in patients (55). Although rare, IFN-α and IFN-β can prompt extensive vascular changes including thrombotic microangiopathy which encompasses endothelial dysfunction, microvascular ischemia, and microangiopathic hemolytic anemia with vascular microaneurysms and stenoses (91).

### Effects of chronically elevated type I interferon signaling in the CNS

Diseases associated with chronically elevated levels of IFN-I in the CNS are collectively termed ‘cerebral interferonopathies’. This diverse group of diseases may be genetic/hereditary (e.g., Aicardi-Goutières Syndrome (AGS), ISG15 deficiency, and USP18 deficiency), autoinflammatory [e.g., systemic lupus erythematosus (SLE) with neurological manifestation], caused by congenital and chronic viral infections (e.g., infections with *Toxoplasma gondii*, rubella virus, cytomegalovirus, herpes simplex virus, hepatitis B and C virus, and human immunodeficiency virus), or without known etiologies such as Degos disease (156–158). Given their many shared symptoms and pathological features, cerebral interferonopathies provide valuable insights into the long-term biological effects of increased IFN-I signaling in the CNS.

AGS is the commonly exemplified cerebral interferonopathy whereby mutations in genes involved in nucleic acid detection and metabolism lead to increased intrathecal IFN-α production (159, 160). So far, mutations in nine genes have been identified to cause AGS: *TREX1*, *RNASEH2A*, *RNASEH2B*, *RNASEH2C*, *SAMHD1*, *ADAR1*, *IFIH1*, *LSM11*, and *RNU7-1* (26, 161). It is proposed that loss-of-function mutations in TREX1, RNASEH2, and SAMHD1 lead to the accumulation of immunostimulatory nucleic acid species derived from endogenous retroviral element expression which activate sensors that induces the expression of IFN-Is (162). Similarly, loss of function in ADAR1 results in lack of posttranscriptional modification of endogenous retroviral element transcripts, resulting activation of MDA5, PKR, and ZBP1, which induces IFN-Is and cell death (163–165). Gain-of-function mutations in *IFIH1* cause an overactive gene product, MDA5, and consequently abnormal induction of IFN-Is (166). In contrast to aberrant IFN-I induction through sensing or regulating endogenous retroviral elements, mutations in *LSM11* and *RNU7-1* result in disrupted histone packing of DNA leading to the activation of cGAS/STING to induce IFN-Is (161).

**Figure 3:**
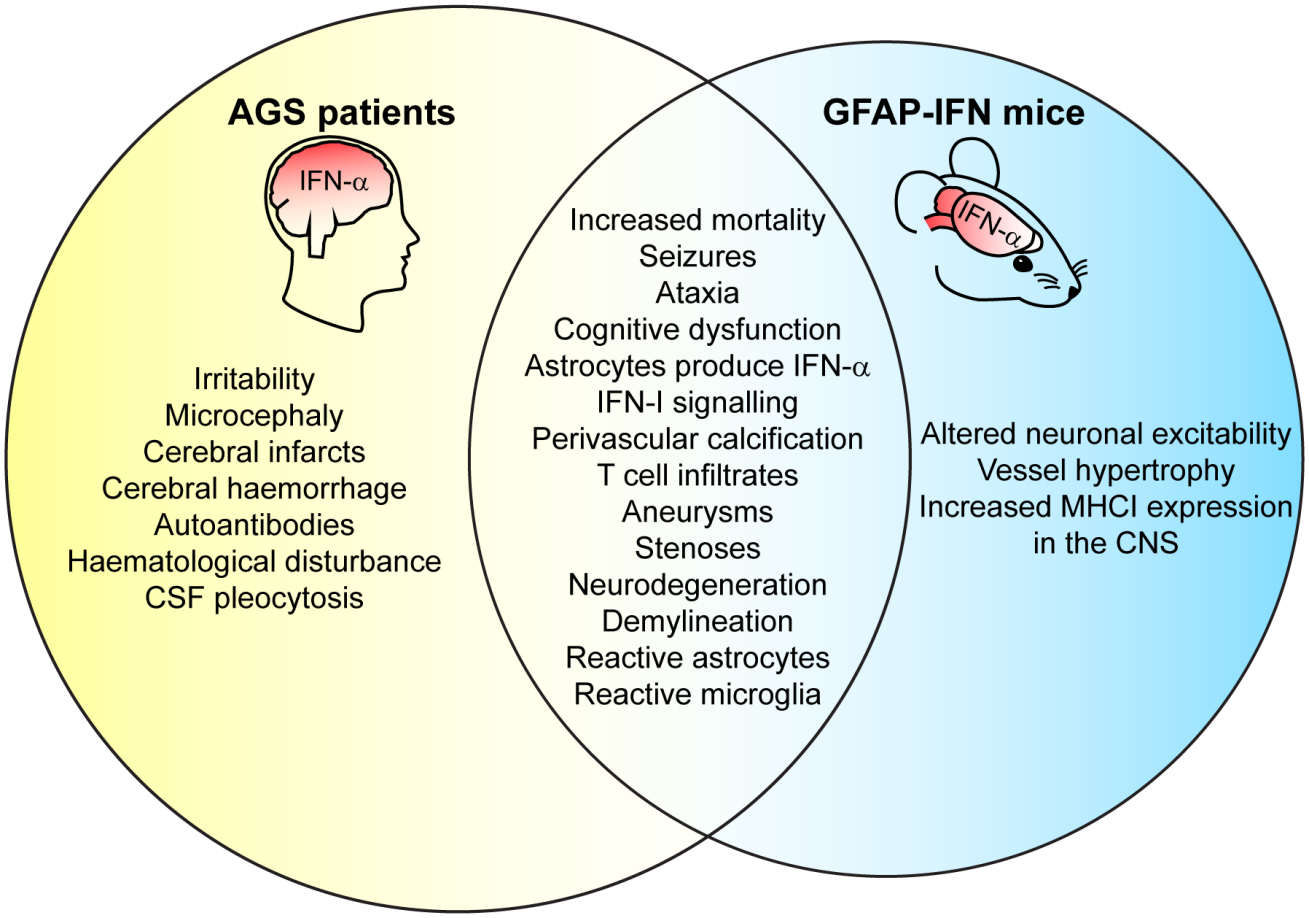
GFAP-IFN mice recapitulate clinical and pathological features of patients with AGS. Venn diagram showing overlap between clinical CNS symptoms and neuropathology observed in the GFAP-IFN mice and patients with AGS. Features that do not overlap and/or have yet to been shown in mice or in patients are also indicated.

Clinically, AGS has an early onset that mimics transplacental-acquired infections and includes increased mortality before adulthood, irritability, slowed cognitive growth, abnormal movements that develop into ataxia, and epileptic seizures (26, 156, 162, 167, 168). Neuroimaging reveals features including microcephaly, white matter disease, intracranial calcification, necrosis, and vasculopathy with stenosis, moyamoya (small and inadequate vessels formed due to the narrowed cerebral artery), aneurysms, infarcts, and hemorrhage (26, 162, 167, 168). Neuropathological brain examinations show demyelination, perivascular calcification, T-cell infiltration, and apoptotic cells (62–64, 169, 170). Consequently, the clinical and neuropathological observations have led to the proposal of AGS being either a leukodystrophy (171, 172) or a microangiopathy (63, 173). Notably, while vessel disease is a common feature in brains from patients with AGS, whether it mediates pathology or is a consequence of disease has not been clarified. Further, immunohistochemistry has revealed that astrocytes are the main source of IFN-α in the CNS in patients with AGS (62–64) and AGS has thus also been classified as an astrocytopathy by some authors (174). Similar to IFN-I therapy, elevated IFN-α plasma and CSF levels correlate with clinical severity in patients with AGS (160). However, there is a lack of knowledge regarding which cell types and molecular mechanisms mediate disease pathology in AGS, a deficit that also extends to other cerebral interferonopathies. This lack of knowledge stems in large parts from the fact that mouse or zebrafish models that mimic the genetic mutations of patients with AGS, do not recapitulate the human disease (175). By contrast, transgenic mice with increased cerebral IFN-I production (GFAP-IFN mice) – recapitulating the one feature common to of all cerebral interferonopathies – develop closely overlapping clinical and pathological changes also present in patients (**Figure 3**) (58, 59).

### Chronic infectious encephalopathy

A key feature of the host immune response to pathogens is the rapid production of IFN-Is that activate and regulate both the innate and adaptive immune response (176). The ultimate aim of this immune response is to limit damage to the host, eliminate the pathogen, and re-establish organismal homeostasis. However, in situations where pathogen elimination is not achieved, chronic production of IFN-Is occurs. This is evident in a range of congenital and chronic infections of the CNS including toxoplasmosis, syphilis, rubella, cytomegalovirus, Zika virus, herpes simplex virus and human immunodeficiency virus (177). Many of the clinical and neuropathological findings mirror those observed in patients with AGS (157) including cognitive and motor dysfunction, microcephaly, leukodystrophy, cerebral calcification, loss of neurons, and gliosis (178, 179) (**Figure 2**). Importantly, these changes are paralleled by elevated cerebral IFN-α levels (180). Further, increased IFN-α levels detected in patients with human immunodeficiency virus are linked to developing neurocognitive disorders (50, 181). Together, these findings indicate a direct association between increased chronic cerebral IFN-I and disease.

### Aging

Aging of the brain concurrently occurs with cognitive decline, reduced neurogenesis, cerebral atrophy, waning of cerebral vascular function, and increased neuroinflammation (182, 183), symptoms which are also seen in patients with cerebral interferonopathies (**Figure 2**). The mechanisms of aging are not well understood and are made more complex by the presence of comorbidities like BBB breakdown (184, 185), dementia, cerebral small vessel disease and neurodegenerative disorders (182, 186). Notably, IFN-β protein and IFN-I signaling are increased in the choroid plexus in the aged CNS of humans and mice (75, 119). Antibody-mediated neutralization of IFNAR1 in mice reversed the aged transcriptomic phenotype while increased IFN-β expression in the choroid plexus of young mice resulted in a transcriptome that reflected that of aged mice (75, 119). Additionally, JAK inhibition reduced cellular senescence and improved physical functions in aged mice (120). Thus, aging and increased IFN-I signaling in the CNS appear to be interlinked, with implications for the further study of age-related cognitive decline.

### Diseases with abnormal protein aggregation

One important and so far, understudied aspect of neurodegenerative diseases is the co-occurrence of inflammation and increased IFN-I signaling. To date, this has probably been best studied in AD. In brain tissue from patients with AD, expression of IFN-Is and IRGs is increased (32, 187, 188), which is supported by similar findings in mouse models of AD (43, 79, 188). A recent study demonstrated that the induction of IFN-I is due to nucleic acid contained in amyloid-beta plaques that stimulates IFN-β production and IFN-I signaling in microglia (43). A role for increased IFN-Is in AD pathogenesis (rather than just being a bystander effect) has been demonstrated in mouse models, where IFNAR1 deletion or neutralization resulted in downregulated expression of proinflammatory cytokines, attenuated microgliosis, increased complement-mediated synapse engulfment, enhanced astrogliosis, and partial improvement in learning (43, 79, 125). Likewise, patients with mild cognitive impairment had increased blood IFN-I signaling compared with healthy controls, which was further increased in those with AD (189). Of note, in a rat model of AD, IFN-β treatment improved memory and reduced inflammatory markers (190), and in humans with subtle cognitive decline, a preclinical feature of AD, reduced blood IFN-I signaling levels is linked to an increased risk of progression to mild cognitive impairment (189). Thus, IFN-Is display protective and damaging properties in AD.

In Parkinson’s disease (PD), increased IFN-Is and IRG products surround Lewy bodies (32, 127, 187), the disease-defining pathological hallmark of PD. Additionally, the protein α-synuclein, that form into Lewy bodies, enhances the signaling of IFN-Is in neurons (126). Ablation of IFNAR1 in a mouse model of PD reduced neuroinflammation and decreased dopaminergic neuronal death (127). The increased IFN-I signaling in the vicinity of protein aggregation, pathological hallmarks of AD and PD, indicates that protein aggregation facilitates localized IFN-I production in surrounding cells. This is supported by studies in mouse models on prion disease, which also involves abnormal protein aggregation. Here, robust IFN-I signaling is seen in microglia (132), and in mice lacking IFNAR1 or STING, disease pathology was delayed (132). Furthermore, increased IFN-I signaling is also observed in the CNS of mouse models of Huntington’s disease (HD) (128, 129, 191) and amyotrophic lateral sclerosis (ALS) (130, 131), other disorders with prominent protein aggregates. Together, these findings suggest that protein aggregates are strong inducers of IFN-I signaling and may contribute to disease progression (**Table 1**).

### Traumatic brain injury

Unlike the previous CNS conditions, traumatic brain injury (TBI) involves external physical disruption to the CNS. Symptoms reflect both trauma severity and impact location and may include depression, memory problems, anxiety, agitation, and motor coordination problems (192, 193). The pathological features around the CNS injury site include necrosis, glial cell activation, BBB leakage, neuron degeneration, neuroinflammation, and leukocyte infiltrates (194), features that also occur in cerebral interferonopathies (**Figure 2**). In response to TBI, chronic local upregulation of IRGs occurs at the injury site, persisting for several months post-injury in both humans and mice (4, 133, 195, 196). Additional increase in IFN-β or IFN-I signaling, for example, in the case of traumatic infection or an aged brain, exacerbates disease outcomes in patients and mice, whilst loss of *Ifnb* and anti-IFNAR1 treatment in mice attenuates the damage from TBI (133, 134, 196–198), further demonstrating the neurotoxic capacity of IFN-Is.

### Trisomy 21

An extra copy of chromosome 21 in humans (trisomy 21) results in diverse symptoms affecting many organs including the CNS. Although symptoms may not all manifest together (9), they include cognitive dysfunction, moyamoya, craniofacial abnormalities, autoimmunity, hematological disorders, intracranial calcification, and early-onset AD (9, 199–203). Some degree of resistance to the development of solid tumors has been observed (9, 199). *IFNAR1* and *IFNAR2* are located on chromosome 21 and their levels are elevated in trisomy 21 (9, 204–206), possibly rendering cells hyperresponsive to IFN-Is. In support, both transcriptomic and proteomic studies of various cell types from trisomy 21 patients show elevated IFN-I signaling and IRG products (9, 204). Notably, many CNS-associated symptoms mirror those observed in cerebral interferonopathies (**Figure 2**) indicating that increased cerebral IFN-Is may contribute to disability in these patients, and trisomy 21 has been suggested to be an interferonopathy by some authors (9, 204). This in turn opens new therapeutic options for patients with trisomy 21 and accordingly, JAK inhibitors, which block formation of the ISGF3 signaling complex, have been used with some success in case studies and mouse models showing improvements in disease (121–124) and is in a clinical trial (ClinicalTrials.gov Identifier: NCT04246372).

### Multiple sclerosis

MS is a demyelinating disease with unclear etiology (207). Patients exhibit a diverse range of symptoms which are largely associated with the location of lesions that occur in the CNS (207). These lesions contain inflammatory leukocytes that presumably mediate oligodendrocyte damage, loss of myelin (208), and local disruption of the BBB (209). IFN-I serum and CSF levels in MS patients do not differ from healthy controls (210). However, there is a focal increase of IFN-I production and IRGs in brain lesions of MS patients and mouse models of MS (71, 211). This mirrors the increase in IFN-I around abnormal protein aggregates and TBI lesions described above, indicating that local production of IFN-I to cellular damage is a common response in the brain. Further, pathological overlaps with AGS/leukodystrophies (212) and MS (**Figure 2**) such as cerebral small vascular disease exist (213).

Although IFN-Is are produced locally in MS and some mouse models, overall, IFN-I signaling appears to be protective. Genetic ablation of IFNAR1 or IFN-β in mice, results in more severe EAE (28). IFN-β is highly effective for the treatment of MS (IFN-α, although effective, is less well tolerated due to adverse effects including increased occurrence of depression) (16, 214, 215). However, the mechanisms by which IFN-Is are beneficial in MS remain unclear and there is variability in the responses to IFN-β, with some MS patients showing improvement, while others having no change or worsening of disease (216, 217). It has been suggested that some MS patients with IFN-I-induced worsening of disease may have been misdiagnosed; MS and neuromyelitis optica spectrum disorder (NMOSD) can cause very similar symptoms, but in contrast to most MS patients, IFN-Is exacerbate disease in NMOSD (218). In addition, variations in responses to IFN-Is could be due to subnormal serum responses to IFN-Is (219, 220). Thus, it is possible that IFN-β treatment rebalances host IFN-I signaling activity in these patients, rather than being excessive or detrimental.

### Therapeutic potential of blocking IFN-I signaling

Currently, there is no cure for cerebral interferonopathies, such as AGS and SLE, and available treatments are primarily aimed at managing symptoms. Treatment is complicated by differences in etiologies, disease progression, severity, and symptoms and importantly by a lack of knowledge regarding the vulnerable and disease-mediating cell types (162). Anti-inflammatory and immunosuppressant drugs (**Figure 1**) such as corticosteroids or methotrexate are often given to dampen inflammation and reduce infiltrating immune cells, while antiepileptics are used to manage seizures (101, 158, 162, 221–223). Careful consideration is required when devising therapeutic strategies as inactivating canonical signaling factors STAT1, STAT2, or IFR9 in GFAP-IFN mice results in exacerbated disease (135–137), demonstrating that maintaining balanced IFN-I signaling is critical.

Recently, targeting the IFN-I signaling pathway has shown some promise. Treatments with anti-interferon, anti-IFNAR, or JAK inhibitors (**Figure 1**, **Table 1**) results in dramatic improvements in some patients with AGS, SLE, and even recovery of patients with peripheral interferonopathies (10, 101–106, 108–112, 221, 224–226). However, these treatments lack support from larger clinical trials, especially in regards to changes in neurological symptoms (162). Importantly, the ability of these treatments to bypass the BBB and improve CNS pathology is yet to be confirmed. Furthermore, the safety profiles of the therapies are noted to include an increased risk of opportunistic infections due to the generalized immunosuppression, as well as an increased risk of major adverse cardiovascular events (227–230). Currently, several clinical trials are underway for patients with AGS (ClinicalTrials.gov Identifier: NCT03921554, NCT04517253, and NCT01724580) and their outcomes will hopefully provide the necessary rationale for the wider use of these treatments. The therapeutic potential of IFN-I signaling inhibition is less clear in the other discussed neurological disorders, with evidence suggesting it may be beneficial in some cases and detrimental in others (**Table 1**).

## Discussion

IFN-Is are a double-edged sword in the CNS. While they are critical for normal brain function and antimicrobial immunity, chronically elevated levels of IFN-Is can be highly neurotoxic. In addition to both the level and signaling duration of IFN-Is, these opposing effects of IFN-Is are in part due to cell-type specific responses, disease-specific contexts, and biological differences between IFN-I subtypes. These parameters modulate the overall tissue response to IFN-Is in the brain. The detrimental effects of IFN-Is are most evident in cerebral interferonopathies which can serve as a paradigm of IFN-I neurotoxicity, providing valuable insight into a broad spectrum of neurological diseases. Recent advancements with single-cell technologies have provided us with a glimpse of the diversity of the IFN-I responses in the CNS. These studies have provided novel insights into the cell-type specificity of the responses to IFN-Is and demonstrated their variability within a single-cell type. Together, this evidence points to a complex coordination to IFN-Is resulting in a highly stimulus- and time-specific response of CNS-resident cells.

## Author contributions

BV wrote the review with revisions by MH. All authors contributed to the article and approved the submitted version.
